# Potential Role of Sugars in the Hyphosphere of Arbuscular Mycorrhizal Fungi to Enhance Organic Phosphorus Mobilization

**DOI:** 10.3390/jof10030226

**Published:** 2024-03-20

**Authors:** Zexing Jin, Guiwei Wang, Timothy S. George, Lin Zhang

**Affiliations:** 1 State Key Laboratory of Nutrient Use and Management, Key Laboratory of Plant-Soil Interactions, College of Resources and Environmental Sciences, Ministry of Education, China Agricultural University, Beijing 100193, China; 15129310266@163.com (Z.J.); gwwang2019@163.com (G.W.); 2 National Engineering Research Center for Efficient Utilization of Soil and Fertilizer Resources, National Engineering and Technology Research Center for Slow and Controlled Release Fertilizers, College of Resources and Environment, Shandong Agricultural University, Taian 271018, China; 3 The James Hutton Institute, Invergowrie, Dundee DD2 5DA, UK; tim.george@hutton.ac.uk

**Keywords:** AM fungi, hyphosphere bacteria, fructose, mycorrhizal pathway, organic P mobilization

## Abstract

Arbuscular mycorrhizal (AM) fungi engage in symbiosis with more than 80% of terrestrial plants, enlarging root phosphorus (P) absorption volume by producing extensive extraradical hyphae (ERH) in the soil. In addition, AM fungi recruit and cooperate with soil bacteria to enhance soil organic P mobilization and improve fungal and plant fitness through hyphal exudates. However, the role of the dominant compounds in the hyphal exudates in enhancing organic P mobilization in the mycorrhizal pathway is still not well understood. In this study, we added sugars, i.e., glucose, fructose, and trehalose, which are detected in the hyphal exudates, to the hyphal compartments (HCs) that allowed the ERH of the AM fungus to grow or not. The results showed that in AM fungus-inoculated pots, adding three sugars at a concentration of 2 mmol C kg^−1^ soil significantly increased the phosphatase activity and facilitated the mobilization of organic P in the HCs. The addition of fructose at a concentration of 2 mmol C kg^−1^ soil was the most efficient in increasing the phosphatase activity and enhancing organic P mobilization. The released inorganic P was then absorbed by the ERH of the AM fungus. The enhanced mobilization of organic P was correlated with the increase in *phoD* gene number and the changing bacterial community in the presence of fungal hyphae. The sugar addition enriched the relative abundance of some bacterial taxa, e.g., Betaproteobacteriales. Our study suggested that the addition of the sugars by mycorrhizae could be a pivotal strategy in managing P uptake in agricultural production, potentially directing future practices to optimize plant–fungi–bacteria interactions for improved P use efficiency.

## 1. Introduction

Arbuscular mycorrhizal (AM) fungi colonize more than 80% of terrestrial plants, including most crops, forming intimate symbiotic relationships [1,2,3]. Inside the roots, AM fungi form arbuscular structures to exchange carbon (C) and phosphorus (P) with plants [4,5]. This interface is connected to a large extraradical hyphae (ERH) network in the soil, which gives the plant a much greater opportunity to explore the soil for P and other resources [6]. The mycorrhizal plants therefore have two pathways to acquire P from soil, i.e., the direct pathway (DP) via root epidermal cells and root hairs and the mycorrhizal pathway (MP) via AM fungal hyphae [7]. In the MP, the ERH of AM fungi can extend up to 13 cm away from the root surface and contribute up to 90% of the total plant P uptake [8,9]. In addition, AM fungi release protons and carboxylates to mobilize less available forms of soil P [10]. Besides the inorganic P, organic P is also an important component of soil P [11]. Especially with the increased application of manure, green manure and other organic fertilizers, the percentage of organic P in agricultural soils is increasing, which often accounts for as much as one-third of the total P in the soil [12,13]. Therefore, it is important to consider how to use the MP to enhance the utilization rate of organic P for more sustainable agricultural systems.

Recent genome sequencing has shown that AM fungi lack genes encoding phytase and other saprotrophic enzymes and therefore have a limited ability to utilize organic P directly by themselves [14,15,16]. Consequently, AM fungi can recruit phosphate-solubilizing bacteria (PSB), which are almost ubiquitous in soil, to cooperate with them to mobilize organic P [5]. In addition, AM fungi have been shown to enhance phosphatase activity released from PSB, leading to better utilization of organic P in the field [5]. During the interaction between AM fungi and bacteria, the hyphal exudates of AM fungi recruit and sustain the bacteria, and the ERH also provide a rapid passage to soil P patches [17]. As such, recent studies show that the core microbiome exists in the hyphosphere (the narrow region of soil around the hyphae where the physical, chemical, and biochemical characteristics are different from the bulk soil) and plays important roles in the mineralization of organic P [18]. The hyphosphere interaction enhances the absorption and transport of P by the ERH of AM fungi, thereafter strengthening the C-P exchange between plants and AM fungi and finally stimulating plant growth and P uptake [19].

Hyphal exudates are the main force to drive the interaction between AM fungi and hyphosphere bacteria [20]. The major components of hyphal exudates are C-rich compounds with low molecular weight [21,22]. Previous studies have used the hair root–AM fungus dual culture system to collect hyphal exudates, identifying sugars such as fructose, glucose and trehalose as the primary components [23]. Sugars in the hyphal exudates provide C sources that increase bacterial activity and stimulate their growth [5]. Additionally, the bacterial strain *Paenibacillus validus* has been found to facilitate the completion of the lifecycle of *Glomus intraradices* even in the absence of plant roots. It has been discovered that the secretion of raffinose by *P*. *validus* can effectively promote the growth of *G*. *intraradices* [24,25].

Soil bacteria exhibit metabolic preferences for different C sources and selectively acquire the substrates from the C source mixtures [26]. For example, it has been found that bacteria enriched in the rhizosphere preferentially consume aromatic organic acids (e.g., nicotinic acid, coumaric acid, salicylic acid, cinnamic acid, and indole-3-acetic acid) secreted by plants [27]. Adding C compounds to simulate root exudates can increase soil nutrient availability by changing the bacterial community structure [28,29,30]. However, the role of specific compounds found in hyphal exudates in affecting bacterial functions in the hyphosphere and their contribution to P acquisition of the MP is less reported. Simulating the addition of fructose in hyphal exudates significantly increased phosphatase activity in the hyphosphere soil and was related to an increase in bacterial population and a shift in bacterial community structure toward species specialized in utilizing fructose for energy metabolism (such as Saccharibacteria) [31]. Each type of bacteria typically possesses relatively specific capabilities for sugar uptake and metabolism, often functioning as part of a group with a collective set of capabilities. Adjustments in the composition of individual sugars can lead to an increase in the abundance of specific bacterial groups. However, due to the widespread cooperation and competition within bacterial communities, such adjustments can result in changes to the overall community structure [27,32]. Knowledge of the diversity and function of the hyphosphere bacteria has increased in recent years due to the development of genomic technology [33]. Among the numerous molecular approaches for assessing bacterial communities, the 16S rRNA gene is universally conserved among all bacteria, serving as a pivotal marker for estimating bacterial abundance [34]. On the other hand, the *phoD* gene, which encodes for alkaline phosphatase, is a key genetic determinant for P solubilization and overall P cycling in soil [35]. Absolute quantification of both the 16S rRNA gene and the *phoD* gene through qPCR provides a useful toolkit for evaluating the function of hyphosphere bacteria.

The aim of this study was to examine the influence of sugars, i.e., glucose, fructose, and trehalose, which are detected in the hyphal exudates, on organic phosphorus mobilization via the mycorrhizal pathway. Understanding the role of these sugars at various concentrations on P availability in the soil will also help advance the regulation of mycorrhizal pathways for plant nutrition. Specifically, we hypothesized that (1) different sugars had different effects on phosphatase activity in the hyphosphere; (2) different sugars lead to different compositions of bacterial communities; and (3) different concentrations of sugars have different effects on the pivotal metabolic pathways of bacteria in the hyphosphere.

## 2. Materials and Methods

### 2.1. Biological Materials and Soil

The seeds of maize (*Zea mays* L., cv. Zhengdan 958) were sterilized with 10% H_2_O_2_ for 15 min and then washed ten times with sterilized deionized water. The seeds were maintained at 27 °C in the dark for 48 h. Then, the seedlings were sown into the pots as described below. The AM fungus used here was *Rhizophagus irregularis* MUCL 43194. The AM fungus was maintained in root organ cultures of carrot (*Daucus carota* L.). The medium containing carrot root segments colonized by *R*. *irregularis* and spores was dissolved with sodium citrate–trisodium citrate buffer (10 mM, pH = 6.0). The mixture was passed through a 30 μm nylon mesh to obtain the root segments and spores. Then, they were washed into a grinder with sterile water and pulverized. The number of spores per mL was determined under the stereoscope. Subsequently, 5 mL of water containing approximately 550 spores was inoculated into each pot. The soil bacterial filtrate was obtained by suspending 100 g of unsterilized fresh soil in 1000 mL of sterile water and filtering through a five-layer quantitative filter paper, which allowed the passage of common soil microbes but efficiently retained spores and hyphae of fungi [36].

A moderately acidic (pH_water_ 6.85; water:soil = 5:1) soil (brown earth soil according to the USDA classification system) collected from Taian, Shandong province, China, was used. The soil contained 0.26 g kg^−1^ total nitrogen, 4.05 g organic matter kg^−1^ soil, 17.43 mg mineral N (NO_3_^−^ and NH_4_^+^) kg^−1^ soil, and 3.87 mg Olsen P kg^−1^ soil (extracted by 0.5 M NaHCO_3_). The soil was dried, sieved, and mixed with 20% (*w*/*w*) river sand and sterilized with 25 kGy ^60^Co γ-radiation to kill the indigenous microbe at the Beijing Radiation Application Research Center. Then, the following nutrients were added to one kg soil: 200 mg N ((NH_4_)_2_SO_4_), 15 mg P (KH_2_PO_4_), 200 mg K (K_2_SO_4_), 50 mg Mg (MgSO_4_·7H_2_O), 5 mg Zn (ZnSO_4_·7H_2_O), 5 mg Mn (MnSO_4_·H_2_O), 2 mg Cu (CuSO_4_·5H_2_O).

### 2.2. Experimental Design

**Figure 1:**
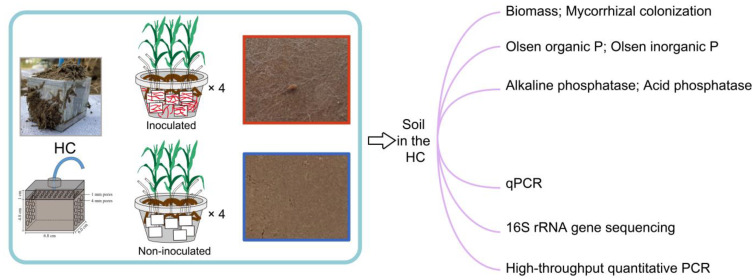
Schematic diagram of the experimental setup and sample collection. Each pot contained seven hyphal compartments (HCs). Half of the pots were not inoculated with *Rhizophagus irregularis* MUCL 43194 spores. After 10 weeks of growth, the plants and HCs in the pots were harvested. These samples were used for the determination of biochemical properties, qPCR, 16S rRNA gene sequencing, and high-throughput quantitative PCR assays to explore the biological and chemical processes involved.

The hyphal compartment (HC) microcosm, developed by Zhang et al. [31], was used to isolate the impact of model exudates on bacterial activity by adding solutions containing the various compounds typical of hyphal exudate (Figure 1). The HC was cuboid (6.8 cm in length, 6.0 cm in width, 5.8 cm in height) and made of PVC board. A hole (6 mm in diameter) was made in the top board of the HC, and a plastic tube (5 mm in diameter, 50 cm in length) was inserted and fixed in position with glue. One centimeter below the top board, another PVC board with holes (1 mm in diameter) was fixed in position and covered with a 30 μm nylon mesh. The solution added from the tube would first arrive at the top nylon mesh and then permeate through to the soil homogeneously. Each HC received 140 g soil containing 100 mg kg^−1^ soil organic P (in the form of phytin, with 19.6% P content, P0410, Tokyo Chemical Industry, Tokyo, Japan). In total, there were eight pots of maize, of which four were inoculated with AM fungi and four were not inoculated with AM fungi. Each pot contained seven different sugar-addition treatments in the HCs: the addition of sterile water, and the addition of three types of sugars at two different concentrations.

The HCs were distributed in the plastic pot with a diameter of 34.5 cm (top) and 24.0 cm (bottom) and a height of 27.0 cm, which contained 9.5 kg of soil in total. Firstly, 3.5 kg of soil was added to the pot and then seven HCs were placed horizontally on the surface of the soil, with the nylon mesh toward the center of the pot. Then, 5.0 kg of soil was added to bury the HCs. The soil was wet up to 70% field capacity by adding 1700 mL of deionized water. Then, the AM fungal inoculants were placed on the soil surface. Five pre-germinated seeds were sown, and 1.0 kg of soil was added to cover the seeds. Seven days after sowing, the seedlings were thinned to three in each pot. During the growth of maize, soil moisture was kept at 18–20% (*w*/*w*, 70% of field moisture capacity) as determined gravimetrically by weighing the pots every 2 days and adding water as necessary. After 1 month post planting, 5 mL soil bacterial filtrate as described above was injected into the HCs through the tubes.

The experiment was conducted in a greenhouse for ten weeks from 4 June to 13 August 2021 with natural light at China Agricultural University, Beijing (116°16′49″ E, 40°1′41″ N). During the 5–9 weeks after sowing, the HC was injected with 20 mL of sterilized water or two different concentrations (14 mmol C L^−1^ and 1.4 mmol C L^−1^) of C solution twice a week, in accordance with the following concentrations in the soil in the HC of each pot. This experiment considered two factors: (1) inoculation (AM fungus-inoculated pots) or not (non-inoculated pots) with AM fungus *R. irregularis* MUCL 43194; (2) different forms of sugar, fructose, glucose, and trehalose at different concentrations of 0, 0.2, and 2 mmol C kg^−1^ soil. The two addition concentrations were determined based on prior research with appropriate adjustments [31]. Each treatment had four replicates. The experiment was arranged in a randomized block design.

### 2.3. Harvest, Determination of Shoot Biomass and Mycorrhizal Colonization

The maize plants were harvested and separated into shoots and roots in each pot ten weeks after sowing. The soil was taken out of the pot and the HCs were untangled from the roots. The soil was gradually removed from the HCs and passed through a 2 mm sieve. Then, the soil was separated into three parts and stored at 4 °C, −20 °C, and −80 °C, respectively, for different analyses. The shoots from three maize plants in each pot were combined into one sample and oven-dried at 105 °C for 30 min to cease metabolic activity and then dried at 65 °C until the weight did not change. Roots were washed with deionized water and then stored at −20 °C. The mycorrhizal colonization of root samples was determined using the method of Trouvelot et al. [37].

### 2.4. Determination of Olsen Inorganic P, Olsen Organic P, and Phosphatase Activity

The Olsen inorganic P was determined according to Olsen et al. [38]: 50 mL of 0.5 M NaHCO_3_ (pH 8.5) was added into the plastic extraction bottle with 2.5 g air-dried soil and was then shaken at a speed of 180 rpm at 25 °C for 30 min. The solution was then filtered. The Olsen inorganic P concentration was determined using the method of molybdenum–antimony and a colorimetric assay at 882 nm. The soil Olsen organic P was determined using the method of potassium persulfate oxidation [39]: 0.15 g of potassium persulfate and 1 mL 0.505 mol L^−1^ H_2_SO_4_ were added to 20 mL of the extracted solution, and digested at 121 °C for 60 min. Then, the total Olsen P was determined by the method of molybdenum–antimony and a colorimetric assay at 882 nm. The content of Olsen organic P was determined by subtracting the amount of Olsen inorganic P from the measured value for total P.

The soil phosphatase activity was determined according to Tabatabai and Bremner [40] with modifications: 0.5 g of soil stored at −20 °C was transferred into a 10 mL centrifuge tube and then 1.6 mL of 200 mM NaHCO_3_ (pH 8.5, for alkaline phosphatase) or CH_3_COONa (pH 5.2, for acid phosphatase) and 0.4 mL *p*-nitrophenyl phosphate (150 mM, Sigma, St. Louis, MO, USA) solution were added. After incubating for 30 min at 30 °C (mixing occasionally), the reaction was terminated by the addition of 2 mL 0.5 M NaOH. The concentration of released *p*-nitrophenyl was determined by a colorimetric assay at 405 nm. The phosphatase activity was expressed as pKatal g^−1^ dry weight (DW) soil.

### 2.5. DNA Extraction and qPCR Analysis of 16S rRNA Gene and phoD Gene

Half a gram of soil stored at −80 °C was used to extract DNA using the FastDNA SPIN Kit for Soil (MP Biomedicals, Santa Ana, CA, USA) following the manufacturer’s instructions. Then, 80 μL of elution buffer was used to elute DNA, which was then quantified using a NanoDrop ND-1000 spectrophotometer (NanoDrop Technologies Inc., Wilmington, DE, USA). DNA was analyzed with real-time q-PCR and prepared for sequencing.

DNA extracted from the hyphosphere soil of each treatment was quantified by real-time q-PCR with 16S rRNA gene- and *phoD* gene-specific primers. The primer sets were 515F (5′-GTGCCAGCMGCCGCGGTAA-3′)/907R (5′-CCGTCAATTCMTTTRAGTTT-3′) and ALPS-F730 (5′-CAGTGGGACGACCACGAGGT-3′)/ALPS-R1101 (5′-GAGGCCGATCGGCATGTCG-3′) [41,42], targeting the V4-V5 region of the 16S rRNA gene and *phoD* gene. TB Green^®^ Premix Ex Taq^TM^ II (Catalog number: RR820A, TaKaRa Bio Inc., Shiga, Japan) was used under the following reaction conditions for the 16S rRNA gene: initial denaturation at 95 °C for 5 min; 40 cycles consisting of denaturation at 95 °C for 15 s; annealing at 60 °C for 30 s; and elongation at 72 °C for 1 min. Fluorescence of SYBR green was detected after every cycle. Then, a dissolution curve was created, while the reaction ended, with 0.5 °C increases from 65 °C to 95 °C. There was no amplification in the negative controls. TB Green^®^ Premix Ex Taq^TM^ II (Catalog number: RR820A, TaKaRa Bio Inc, Shiga, Japan) was used under the following reaction conditions for the *phoD* gene: initial denaturation at 95 °C for 5 min; 39 cycles consisting of denaturation at 95 °C for 15 s; annealing at 60 °C for 30 s; and elongation at 72 °C for 1 min. Fluorescence of SYBR green was detected after every cycle. Then, a dissolution curve was created, while the reaction ended, with 0.5 °C increases from 65 °C to 95 °C. There was no amplification in the negative controls. Before constructing a standard curve for absolute quantification of the 16S rRNA gene and *phoD* gene, the plasmid was sequenced for verification. The standard curve was constructed in triplicate using five serial 10-fold dilutions, and quantification was performed by determining the starting copy number by considering the concentration of the plasmid and number of base pairs.

### 2.6. 16S rRNA Gene Sequencing and Bioinformatics Processing

PCR was performed with a 515F/907R primer, which allowed the analysis of the bacteria and assessment of their community structure. Amplicons were pooled in equal amounts, and pair-end 2 × 300 bp sequencing was performed using the Illlumina MiSeq platform with MiSeq Reagent Kit v3 at Shanghai Personal Biotechnology Co., Ltd. (Shanghai, China) after individual quantification.

Microbiome bioinformatics was performed with QIIME2 according to the official tutorials [43]. Briefly, raw sequence data were demultiplexed using the demux plugin followed by primer cutting with the cutadapt plugin [44]. Sequences were then quality-filtered, denoised, and merged and chimera were removed using the DADA2 plugin [45]. Non-singleton amplicon sequence variants (ASVs) were aligned with mafft [46]. Taxonomy was assigned to ASVs using the classify-sklearn Naive Bayes taxonomy classifier in the feature-classifier plugin [43] against the SILVA Release 132 Database [47]. The bacterial 16S rRNA gene sequences obtained from this study were deposited in the National Center for Biotechnology Information Sequence Reads Archive (SRA accession: PRJNA1050038).

### 2.7. High-Throughput Quantitative PCR

We used high-throughput quantitative PCR to investigate the abundance and diversity of the soil nutrient cycling genes of the hyphosphere soil samples by the Wafergen SmartChip Real-time PCR system (WaferGen Biosystems, Fremont, CA, USA) [48]. The protocol of high-throughput quantitative PCR was conducted under the following reaction conditions: initial denaturation at 95 °C for 10 min, followed by 40 cycles of denaturation at 95 °C for 30 s, annealing at 58 °C for 30 s, and extension at 72 °C for 30 s. Then, we analyzed the results by using SmartChip qPCR software (version 4.9) and excluded the results with multiple amplification efficiencies or melting peaks beyond the range of 80–120%. The data from any threshold cycle (CT) below 31 were used for further analysis. The relative gene copy number of all targeted genes was calculated according to Looft et al. [49]. Relative gene abundance was defined as the proportion of the abundance of a functional gene to the abundance of the 16S rRNA gene [50].

### 2.8. Statistical Analyses

All data were checked for normality using Kolmogorov–Smirnov tests and homogeneity of variances using Levene’s tests with IBM SPSS Statistics 19. Significant differences (*p* ≤ 0.05) among different treatments were evaluated by Tukey’s HSD test. The method of linear regression was used to model and test the correlation between 16S rRNA gene copy number and ALP activity, the correlation between *phoD* gene copy number and ALP activity, and the correlation between the relative abundance of genera and ALP activity.

Three microbial co-occurrence networks were established for the three sugar-addition treatments. A Spearman’s correlation between two ASVs was considered statistically robust if the Spearman’s correlation coefficient (ρ) > 0.65 and the *p* value < 0.01. All the robust correlations identified from pairwise comparison of the ASVs abundance form a correlation network where each node represents one ASV, and each edge stands for a strong and significant correlation between the nodes. Networks were visualized using the R package “igraph”. All statistics analyses were performed in R (version 4.1.1) unless otherwise indicated.

To address the potential systemic effects inherent in our experimental design, we constructed a mixed-effects model to account for random effects to assess the systemic impact. To establish these models, we treated the response variables—ALP activity, ACP activity, concentrations of Olsen inorganic P, Olsen organic P, and gene copy numbers of 16s rRNA and *phoD* (both log-transformed)—as a combination of fixed and random effects. Essentially, the response variable in each model was conceptualized as the outcome influenced by both fixed effects and random effects. For fixed effects, we considered the fungal inoculation and the seven different concentrations of carbon addition as our main effects. They are termed ‘fixed’ because the levels of these factors are predetermined by the researcher and are applied to the experimental subjects. Regarding random effects, we focused on the potential non-independence of HCs within each of the pots. If the values of the random effects were small, it implied that the differences between the HCs were not random but rather could be well predicted by the fixed-effects once they were accounted for. This indicated that the HCs were relatively independent of each other. The differences between the treatments were compared by Tukey’s HSD test (*p* < 0.05).

## 3. Results

### 3.1. Mycorrhizal Colonization and Maize Shoot Dry Biomass

In the AM fungus inoculation treatment, mycorrhizal colonization was 66.83% (Figure S1a); in fact, the hyphal network was visible on the surface of the soil section in the HCs (Figure 1). In the non-AM fungus inoculation treatment, the mycorrhizal colonization was nearly zero (Figure S1a). The surface of the soil section in the HCs did not show visible hyphae (Figure 1). In the non-AM fungus inoculation treatment, the maize shoot biomass was 13.94 g, while in the AM fungus inoculation treatment, the maize shoot biomass was 25.98 g (Figure S1b).

### 3.2. The Influence of AM Fungal Presence and Sugar Addition on the Phosphatase Activity and Olsen P of the Soil in the HCs

**Figure 2:**
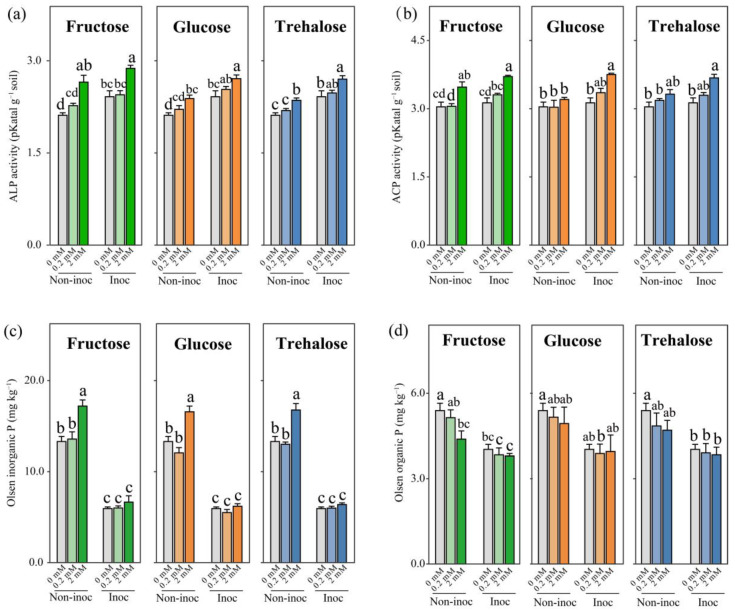
The soil of hyphal compartments (HCs) was tested for (**a**) alkaline phosphatase activity (ALP), (**b**) acid phosphatase activity (ACP), (**c**) Olsen inorganic P, and (**d**) Olsen organic P following the supplementary addition of 0, 0.2, and 2 mmol C kg^−1^ soil of fructose, glucose, and trehalose to the HCs in AM fungus-inoculated or non-inoculated pots. Bars are means ± SEs (*n* = 4). Different lowercase letters indicate significant differences among the treatments (Tukey’s HSD, *p* < 0.05). Non-inoc, non-inoculated pots. Inoc, AM-fungus inoculated pots.

The addition of 2 mmol C kg^−1^ soil of all three sugars to the HCs significantly increased the alkaline phosphatase activity regardless of whether in AM fungus-inoculated or non-inoculated pots. In AM fungus-inoculated pots, compared to the non-inoculated pots, the HCs showed an increasing trend in the activities of both acid phosphatase and alkaline phosphatase in all three sugar-addition treatments (Figure 2a,b). In non-inoculated pots, the 2 mmol C kg^−1^ soil sugar addition significantly increased the soil Olsen inorganic P content compared with 0 or 0.2 mmol C kg^−1^ soil sugar addition to the HCs (Figure 2c). In AM fungus-inoculated pots, the soil Olsen inorganic P showed no significant difference among the different sugar additions to the HCs (Figure 2c). Furthermore, under the same amount of sugar addition, there was a significant reduction in the content of Olsen inorganic P in HCs when AM fungus was inoculated, as well as a decreasing trend in Olsen organic P (Figure 2c,d).

**Figure 3:**
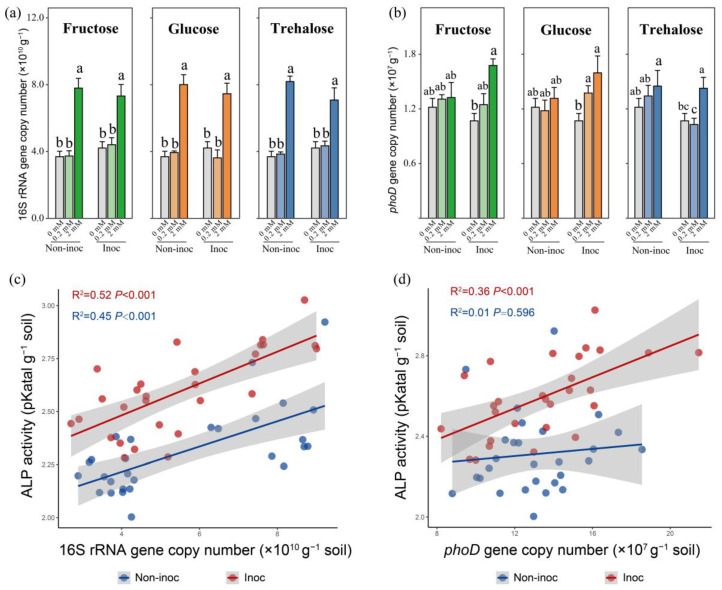
Copy number of (**a**) 16S rRNA gene and (**b**) *phoD* gene in three types of sugar addition to the HCs in AM fungus-inoculated or non-inoculated pots. Bars are means ± SEs (*n* = 4). Different lowercase letters indicate significant differences among the treatments (Tukey’s HSD, *p* < 0.05) (**c**) The correlation between 16S rRNA gene copy number and alkaline phosphatase activity (ALP) (R^2^ = 0.52, *p* < 0.001, AM fungus inoculated; R^2^ = 0.45, *p* < 0.001, non-inoculated). (**d**) The correlation between *phoD* gene copy number and ALP (R^2^ = 0.36, *p* < 0.001, AM fungus inoculated; R^2^ = 0.01, *p* < 0.596, non-inoculated). Non-inoc, non-inoculated pots. Inoc, AM fungus-inoculated pots.

The addition of 2 mmol C kg^−1^ soil sugar to the HCs significantly increased the 16S rRNA gene copy number in AM fungus-inoculated or non-inoculated pots (Figure 3a). Moreover, the increase in the copy number induced by the three forms of sugars was about 2-fold (Figure 3a). In non-inoculated pots, whatever the form of sugar or the added concentration to the HCs, there was no significant change in the *phoD* gene copy number (Figure 3b). In AM fungus-inoculated pots, 2 mmol C kg^−1^ soil sugar addition to the HCs led to an increase in the *phoD* gene copy number compared to 0 or 0.2 mmol C kg^−1^ soil sugar addition (Figure 3b). In non-inoculated pots, only the 16S rRNA gene copy number (R^2^ = 0.45, *p* < 0.001) was significantly positively correlated with the alkaline phosphatase activity (Figure 3c,d). In AM fungus-inoculated pots, both the 16S rRNA gene copy number (R^2^ = 0.52, *p* < 0.001) and the *phoD* gene copy number (R^2^ = 0.36, *p* < 0.001) were significantly positively correlated with the alkaline phosphatase activity (Figure 3c,d).

### 3.3. The Influence of Sugar Additions on the Bacterial Communities 

**Figure 4:**
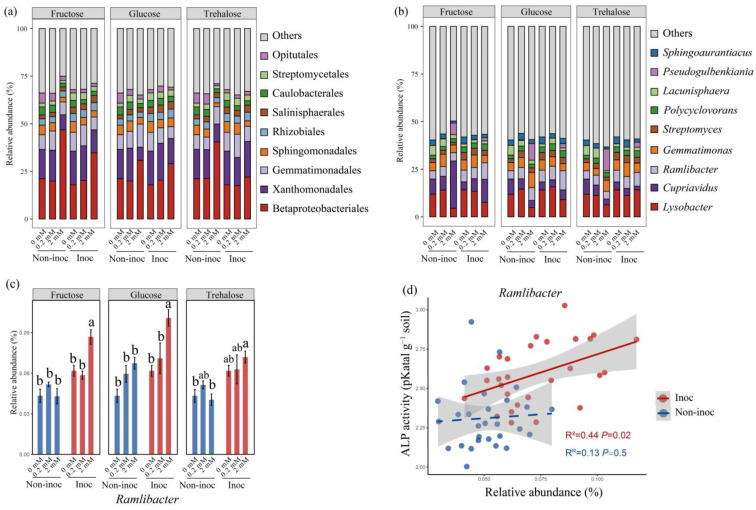
Relative abundance (%) of the 9 most abundant bacterial (**a**) orders and (**b**) genera in AM fungus-inoculated or non-inoculated pots, following the addition of 0 mmol C kg^−1^ soil, 0.2 mmol C kg^−1^ soil, and 2 mmol C kg^−1^ soil sugars to the HCs. (**c**) The relative abundance of the genera *Ramlibacter* across treatments. Bars are means ± SEs (*n* = 4). Different lowercase letters indicate significant differences among the treatments (Tukey’s HSD, *p* < 0.05). (**d**) The correlation between the relative abundance of *Ramlibacter* and alkaline phosphatase activity (ALP) (R^2^ = 0.44, *p* = 0.02, AM fungus inoculated; R^2^ = 0.13, *p* = 0.5, non-inoculated). Non-inoc, non-inoculated pots. Inoc, AM fungus-inoculated pots.

After the addition of the three sugars at 2 mmol C kg^−1^ soil, some orders (i.e., Betaproteobacteriales increased from 21.19% to 30.83–46.88%, Xanthomonadales decreased from 15.38% to 6.9–9.33%) or genera (i.e., *Pseudogulbenkiani* increased from <0.02% to 6.11–11.46%, *Lysobacter* decreased from 11.90% to 4.57–6.31%) increased or decreased in relative abundance, respectively (Figure 4a,b). After analyzing the correlations between the nine most abundant genera and the phosphatase activity, we ultimately discovered that in AM fungus-inoculated pots, only the genus *Ramlibacter*, which belongs to the Betaproteobacteriales order, showed a positive correlation with alkaline phosphatase activity in the HCs (Figure 4c,d). In non-inoculated pots, the relative abundance of the genus *Ramlibacter* had no correlation with alkaline phosphatase activity in the HCs (Figure 4c,d). Specifically, we found that with an addition of fructose at 2 mmol C kg^−1^ soil to the HCs, the abundance of the genus *Cupriavidus* was significantly greater compared to the addition of glucose and trehalose at 2 mmol C kg^−1^ soil to the HCs in non-inoculated pots (Figure 4b, *n* = 4, Tukey’s HSD, *p* < 0.05).

**Figure 5:**
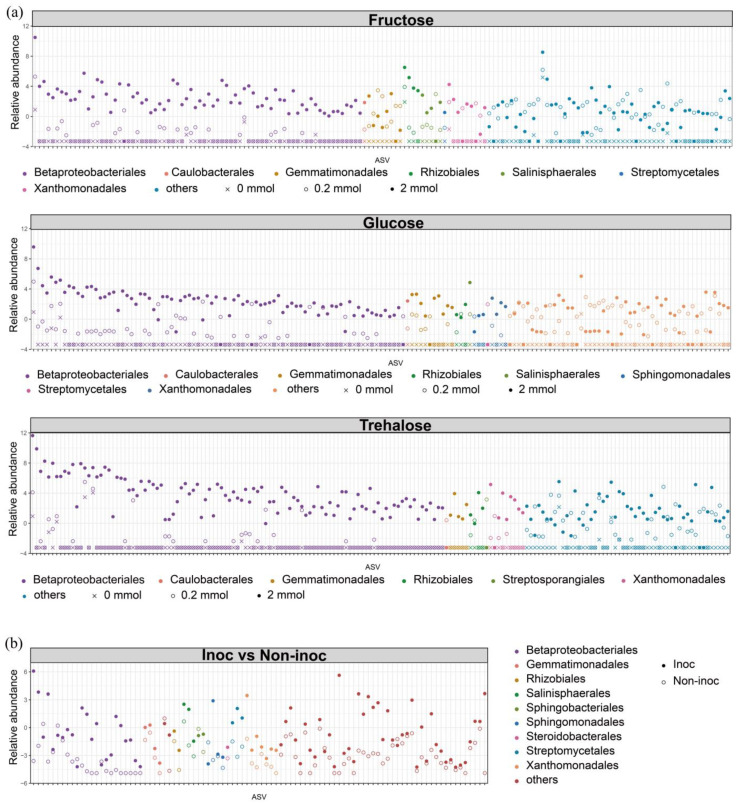
(**a**) The abundance of enriched ASVs corresponding to the addition of fructose, glucose, and trehalose at concentrations of 2 mmol C kg^−1^ soil, 0.2 mmol C kg^−1^ soil, and 0 mmol C kg^−1^ soil to the HCs in non-inoculated pots. The color of the points represents the order-level classification, while the shape of the points represents the sugar addition concentration. The abundance of ASVs was normalized using DeSeq2. (**b**) Abundance of ASVs in the HCs between AM fungus-inoculated pots and non-inoculated pots. The color of the points represents the order-level classification, while the shape of the points represents the AM fungus being inoculated or not. The abundance of ASVs was normalized using DeSeq2. ASVs with significant log2-fold change (DESeq2: *p* < 0.05) were identified as enriched ASVs. Non-inoc, non-inoculated pots. Inoc, AM fungus-inoculated pots.

The enriched ASVs of bacteria after the addition of different concentrations of sugars to the HCs in non-inoculated pots were calculated. The number of ASVs enriched with 2 mmol C kg^−1^ soil sugar addition to the HCs was larger than that of 0.2 mmol C kg^−1^ soil sugar addition (Figure S2). The ASVs enriched by each sugar at both concentrations added to the HCs (i.e., 0.2 and 2 mmol C kg^−1^ soil) were combined and their abundances were standardized. These enriched ASVs mainly belonged to the orders of Betaproteobacteriales, Caulobacterales, Gemmatimonadales, and Rhizobiales in all three sugar-addition treatments (Figure 5a). In addition, the enriched ASVs had the greatest abundance mainly at 2 mmol C kg^−1^ soil, intermediate abundance at 0.2 mmol C kg^−1^ soil, and the smallest abundance at 0 mmol C kg^−1^ soil (Figure 5a). These ASVs showed a gradual increase in relative abundance after sugar addition to the HCs. The enriched ASVs were compared between the treatments in AM fungus-inoculated and non-inoculated pots. The enriched ASVs in the HCs mainly belonged to the order Betaproteobacteriales and their relative abundance were greater in AM fungus-inoculated pots (Figure 5b).

**Figure 6:**
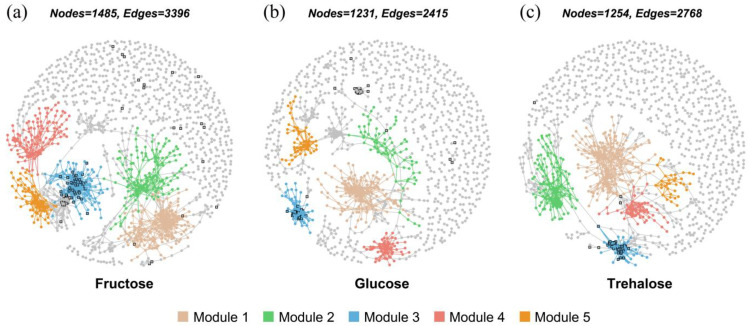
Co-occurring network of bacterial ASVs and in (**a**) fructose, (**b**) glucose, and (**c**) trehalose addition treatments. Each sugar network included all three added concentrations (0, 0.2, and 2 mmol C kg^−1^ soil) and two AM fungus-inoculated conditions. A Spearman’s correlation between two ASVs was considered statistically robust if the Spearman’s correlation coefficient (ρ) >0.65 and the *p* value < 0.01. All the robust correlations identified from pairwise comparison of the ASV abundance form a correlation network where each node represents one ASV, and each edge stands for a strong and significant correlation among the nodes. The square with the black border represents the enriched ASVs of bacteria after the addition of corresponding sugars to the HCs in non-inoculated pots.

Three co-occurring networks were constructed and each sugar network included all three added concentrations (0, 0.2, and 2 mmol C kg^−1^ soil) and two AM fungus-inoculated conditions. The co-occurring network of fructose had the greatest number of nodes, edges, and average connectivity (Figure 6, Table S1). The top five network modules of three co-occurring networks are shown and the enriched ASVs are primarily clustered in module 3 of the three networks (Figure 6).

### 3.4. The Influence of AM Fungal Presence and Sugar Additions on the Functional Genes Related to C Degradation, C Sequestration, N Metabolism, and P Metabolism

**Figure 7:**
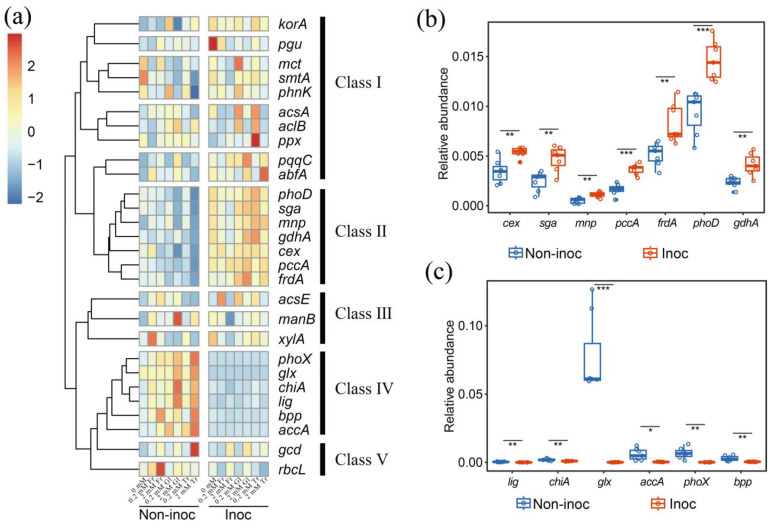
(**a**) Heatmap of SmartChip profile; relative abundance of C and P turnover functional genes in all treatments. Genes were clustered into 5 classes (class I–V) based on the similarity of their abundance across different treatments. Fr: fructose; Gl: glucose; Tr: trehalose. (**b**) The comparison of the relative abundance of genes in class II in AM fungus-inoculated and non-inoculated pots. Each dot represents one cell in the heatmap, referring to the mean (*n* = 4) of the relative abundance of genes in a single treatment. (**c**) The comparison of the relative abundance of genes in class IV in AM fungus-inoculated and non-inoculated pots. Each dot represents one cell in the heatmap, referring to the mean (*n* = 4) of the relative abundance of genes in a single treatment. Non-inoc, non-inoculated pots. Inoc, AM fungus-inoculated pots. Different asterisks indicate significant differences between the treatments (*t*-test, *p* < 0.05 (*), *p* < 0.01 (**), *p* < 0.001 (***)).

In AM fungus-inoculated pots, the relative abundance of the alkaline phosphatase gene (*phoD*) and glucose dehydrogenase gene (*gdhA*) was increased in the HCs. The relative abundance of C degradation genes (*sga*, *mnp*, *cex*) and C sequestration genes (*pccA*, *fdrA*) was also increased (Figure 7a,b). The relative abundance of the alkaline phosphatase gene (*phoX*), C degradation genes (*lig*, *chiA*, *glx*), C sequestration gene (*accA*), and P-cycling gene (*bpp*) was decreased in the HCs (Figure 7a,c). Additionally, in non-inoculated pots, the addition of 2 mmol C kg^−1^ soil of fructose to the HCs increased the relative abundance of N cycle-related genes, such as *nifH*, *nirK1*, *nirK2*, *nosZ1*, *nirS1*, and *nirS2*, as well as C sequestration genes (*rbcL*, *acsE*). (Figure S3). In contrast, 2 mmol C kg^−1^ soil glucose addition to the HCs increased the relative abundance of genes involved in the P cycle, e.g., the pyrroloquinoline–quinone synthase gene *pqqC* and the genes related to C degradation (*manB*, *lig*, *chiA*), carbon sequestration (*aclB*), and the N cycle (*napA*) (Figure S3). Furthermore, 2 mmol C kg^−1^ soil trehalose addition to the HCs increased the relative abundance of the alkaline phosphatase gene (*phoX*), quinoprotein glucose dehydrogenase gene (*gcd*), organic P mineralization gene (*bpp*) in the P cycle, C degradation gene (*glx*), and N cycle gene (*nirS3*) (Figure S3).

## 4. Discussion

The hyphal exudates of AM fungi are potentially key factors driving biological interaction to mobilize organic P in the hyphosphere. In this study, we simulated three model exudates with different concentrations of sugars (i.e., glucose, fructose, and trehalose) detected in the hyphal exudates to regulate the hyphosphere interaction to enhance organic P mobilization of the MP. We found that in the absence of AM hyphae in the soil, the addition of relatively large amounts of all the sugars led to increased phosphatase activity and the conversion of organic P to inorganic P, which accumulated (Figure 2). Importantly, while this impact of model exudates was associated with a significant increase in the 16S rRNA gene copy number, it was not related to the increased number of *phoD*, a gene known to be important in the production of phosphatase (Figure 3). Moreover, in the presence of AM fungi, the enhanced mobilization of organic P was associated with changes not only in the bacterial abundance (16S rRNA gene) but also in the proportion of the *phoD* gene in the hyphosphere, which was not the case in the absence of AM fungi (Figure 3). Such changes specifically occurred in certain taxa, such as Betaproteobacteriales. Additions of three forms of sugars all resulted in a stepwise increase in the abundance of a large number of ASVs within the Betaproteobacteriales, which formed tightly knit clusters in co-occurring networks. Our study therefore provides a new insight into the hyphosphere regulation of organic P mobilization of the MP.

It should be noted that our experimental setup used a nested design, where the HCs within the same pot may have potential systematic errors. To address this, we calculated the random effects based on a mixed-effects model to assess the influence of factors other than the experimental setup, aimed at evaluating the possibility of interference between HCs. We determined that the primary influence on the indicators—ALP, ACP, Olsen inorganic P, Olsen organic P, and the gene copy numbers of 16s rRNA and *phoD*—resulted from fixed effects, which in the context of our experiment pertained to inoculation or not, as well as sugar concentration. The fixed effects were responsible for 99%, 98.7%, 98.4%, 94.4%, 99.1%, and 70.7% of the variability observed in these respective indicators, demonstrating that the experimental variables used in our study were the main factors affecting the outcomes. Additionally, although the soil in the root compartments of inoculated and non-inoculated treatments could differ, this study focused on the regulation within the HCs, reflecting regions dominated by AM fungi activity that were inaccessible to the root system in actual conditions. Each HC was inoculated with an identical soil bacterial suspension to ensure uniform conditions across all treatment groups. From the beginning of the experiment, the variables were the allowance or restriction of AM fungal hyphae penetration and carbon addition. Prior to harvesting the HC, the outermost 1 cm of soil was removed to eliminate potential external environmental influences.

### 4.1. The Addition of Sugars Enhanced Organic P Mobilization for the Mycorrhizal Pathway

Photosynthesis-derived C sources are distributed by plants via AM fungal hyphae, impacting the biogeochemical cycle of different elements, especially P, in the hyphosphere [51,52]. Arbuscular mycorrhizal fungi release photosynthetic C into the hyphosphere in the form of hyphal exudates, which are comprised of amino acids, carbohydrates, carboxylates, nucleoids, proteins, and signaling molecules [52,53]. However, how sugars regulate the hyphosphere biological interaction to improve the organic P mobilization for the MP is less understood. In the present study, we found that when we added three forms of sugar at a concentration of 2 mmol C kg^−1^ the alkaline phosphatase was increased, which correspondingly increased Olsen inorganic P in the soil. These results suggest that the addition of sugars at a larger concentration enhanced soil organic P mineralization; however, without the presence of the fungus, this was not associated with *phoD*, but with the upregulation of other phosphorus cycling genes. Most bacteria in the soil are dormant, and the introduction of external carbon sources can prime their activities. Low concentrations of carbon can only satisfy the basic metabolism of bacteria, while high concentrations of carbon are necessary for their proliferation. This study found that the addition of sugar at 2 mmol C kg^−1^ soil caused a significant effect. Furthermore, we observed an increase in the absolute copy numbers of the bacterial 16S rRNA gene, indicating that the high concentration of carbon in this study led to an increase in the overall absolute number of bacteria, which may result in a significant effect.

The *phoD* gene serves as a principal indicator gene for the synthesis of phosphatases and reflects the potential for the mobilization of organic P [35]. In the present study, we noted a strong positive correlation between the *phoD* gene copy number and alkaline phosphatase activity. In AM fungus-inoculated pots, 2 mmol fructose addition to the HCs resulted in the greatest *phoD* gene copy number and thus the greatest alkaline phosphatase activity, suggesting that, once recruited, the *phoD*-producing bacteria were able to thrive in the high-C-addition treatments. However, the addition of fructose and other sugars at smaller concentrations to the HCs, i.e., 0.2 mmol C kg^−1^ soil, did not change the phosphatase activity, bacterial abundance, or *phoD* copy number, even in AM fungus-inoculated pots. This observation corroborated previous research findings [31]. Collectively, our results suggest that adding a large concentration of fructose in the presence of AM fungal hyphae was the most efficient approach to enhance the organic P mobilization for the MP.

### 4.2. The Addition of Sugars Changed Bacterial Communities in the Hyphosphere

Besides the *phoD* gene copy number, the structure of bacterial communities is another factor influencing the organic P mobilization in the hyphosphere. This influence is largely attributed to the exudates from AM fungal hyphae, which are known to shape specific bacterial communities and modulate their roles in the turnover of organic P [18,20]. Previous research revealed that such exudates alter the composition of soil bacterial communities involved in organic N and P mineralization [5,54]. The addition of 2 mmol C kg^−1^ soil of different sugars had different effects on the bacterial community and the P mineralization function of soil. Adding 2 mmol C kg^−1^ soil of sugars also had a significant enrichment on the core microbiota of the hyphosphere (mainly enriched ASVs from Betaprobacteriales). The core microbiota may possess greater alkaline phosphatase enzyme activity. Adding 2 mmol C kg^−1^ soil of fructose increased the bacterial network complexity compared with the other sugars. It showed that the fructose addition encouraged a broader establishment of connections among more bacteria. These results suggested that adding 2 mmol C kg^−1^ soil of fructose may shift the functional specificity of soil bacteria toward P mobilization (Figure 6).

### 4.3. The Effect of the Addition of Sugars on Changing the C-, N-, and P-Related Functional Genes of Bacteria in the Hyphosphere

In addition to changing the abundance and community structure of bacteria, we studied the influence of adding sugars on the C-, N-, and P-related functional genes of bacteria in the hyphosphere. Adding 2 mmol C kg^−1^ soil of fructose notably increased the relative abundance of N cycle-related genes, including *nifH* and various nitrogen oxide reductases (*nirK1*, *nirK2*, *nosZ1*, *nirS1*, *nirS2*), which highlighted the potential of fructose in steering N cycling within the bacterial community, in addition to P. The C from these sugars provides the building blocks for cellular biomass, while energy derived from their metabolism is used to fuel various cellular processes, including the assimilation of N. In contrast, an equivalent concentration of glucose resulted in a shift favoring P cycle enrichment, particularly increasing the abundance of genes such as *pqqC*, which is essential in synthesizing a cofactor required for enzymes in phosphate solubilization and C degradation genes (*manB*, *lig*, *chiA*). This indicated that glucose availability might inform a P cycle-centric bacterial resource allocation [55]. Easily metabolized sugar activates soil bacteria, increases their proliferation, and promotes biological mineralization and their enzymatic capabilities to break down more complex and recalcitrant materials. Furthermore, the supply of 2 mmol C kg^−1^ soil of trehalose was linked to an increase in the *phoX* gene among others, suggesting that this sugar could affect the alkaline phosphatase activity of bacteria. The sugar preference is typically a result of the efficiency of transport and metabolism processes, which can confer a selective advantage for survival and reproduction [32]. Bacterial sugar preferences can be complex and are rooted in their metabolic pathways, environmental adaptation, and evolutionary history. In this study, two methods were used to assess the gene abundance: one measured absolute abundance, while the other measured relative abundance. Absolute abundance reflects the actual number of gene copies, which correlates well with phosphatase activity. Relative abundance, as measured by high-throughput quantitative PCR, is defined as the proportion of the abundance of a functional gene to the abundance of the 16S rRNA gene, which represents the quantity of bacteria. The relative abundance of C, N, and P genes in this study indicates the ratio of gene copies in relation to the bacterial count, or in other words, the gene abundance per unit of bacterial quantity. This represents the proportion of bacteria that possess these functional genes or have a large copy number of these genes in the bacterial community. Bacteria with larger gene copy numbers may potentially exhibit stronger functionality for that gene [56].

### 4.4. Conclusions

Here, we demonstrated that the addition of carbon in the form of a range of sugars typical of the hyphal exudates in a large concentration increased the activity of phosphatase and promoted the mineralization of organic P to inorganic forms. The increased phosphatase activity and organic P turnover in sugar-treated soil in the absence of AM fungi was related to the selection of particular groups of bacteria and likely associated with increased bacterial abundance and activity and concomitant increases in biological mineralization and upregulation of P-cycling genes other than *phoD*. These results suggest that the promotion of the mycorrhizal pathway for sustainable use of soil P could be achieved by enhancing the AM fungi’s ability to both deliver sugar to the hyphosphere in their exudates and recruit *phoD*-expressing bacteria, and then acting as a sink for the inorganic P mineralized. The ASVs enriched by the three model exudates were all concentrated within the same order, Betaproteobacteriales mirrors the uniformity in the regulation of the principal carbohydrate exudates by AM fungi and also demonstrates the absence of conflict in the control of the three major model exudates secreted by AM fungi under actual conditions. These enriched ASVs clustered in the same module of the co-occurrence network, further illustrating that these ASVs were not acting in isolation but as a functional group exerting their effects simultaneously. This implied that the abundance of ASVs and the interactions between them ultimately led to the functional profile.

## Data Availability

The sequences generated in this study are available in NCBI GenBank under the accession number PRJNA1050038.

## Supplementary Materials

The following supporting information can be downloaded at https://www.mdpi.com/article/10.3390/jof10030226/s1. Figure S1: (a) Mycorrhizal colonization of the soil in hyphal compartments in AM fungus-inoculated pots. (b) Shoot biomass of maize plants in AM fungus-inoculated or non-inoculated pots. The asterisks indicate significant differences between the two treatments (*t*-test, *p* < 0.001); Figure S2: Venn plot showing the number of ASVs enriched in the hyphal compartments upon the three sugars’ addition with two concentrations compared to non-sugar addition in non-inoculated pots. Green represents the number of ASVs enriched at 0.2 mmol C kg^−1^ soil compared to 0 mmol C kg^−1^ soil. Red represents the number of ASVs enriched at 2 mmol C kg^−1^ soil compared to 0 mmol C kg^−1^ soil. Orange represents the number of the same ASVs enriched at both 0.2 mmol C kg^−1^ soil and 2 mmol C kg^−1^ soil; Figure S3: Heatmap of SmartChip profile, relative abundance of carbon (C), nitrogen (N), and phosphorus (P) turnover functional genes in the hyphal compartments upon three forms of sugar addition with three added concentrations (0, 0.2, 2 mmol C kg^−1^ soil) in non-inoculated pots. The values shown represent the mean relative abundance (*n* = 4) of genes in the respective sugar addition. Fr: fructose; Gl: glucose; Tr: trehalose; Table S1: The topological properties of co-occurrence networks in hyphal compartments after the addition of three forms of sugars; Table S2: Classification of functional genes.

## References

[1] Bonfante P., Genre A. (2010). Mechanisms underlying beneficial plant-fungus interactions in mycorrhizal symbiosis. Nat. Commun..

[2] Smith S.E., Smith F.A. (2011). Roles of arbuscular mycorrhizas in plant nutrition and growth: New paradigms from cellular to ecosystem scales. Annu. Rev. Plant Biol..

[3] van Der Heijden M.G.A., Martin F.M., Selosse M.A., Sanders I.R. (2015). Mycorrhizal ecology and evolution: The past, the present, and the future. New Phytol..

[4] Walder F., van Der Heijden M.G.A. (2015). Regulation of resource exchange in the arbuscular mycorrhizal symbiosis. Nat. Plants.

[5] Zhang L., Xu M., Liu Y., Zhang F., Hodge A., Feng G. (2016). Carbon and phosphorus exchange may enable cooperation between an arbuscular mycorrhizal fungus and a phosphate-solubilizing bacterium. New Phytol..

[6] Miller R.M., Jastrow J.D., Reinhardt D.R. (1995). External hyphal production of vesicular-arbuscular mycorrhizal fungi in pasture and tallgrass prairie communities. Oecologia.

[7] Shi J., Wang X., Wang E. (2023). Mycorrhizal symbiosis in plant growth and stress adaptation: From genes to ecosystems. Annu. Rev. Plant Biol..

[8] Li X.L., George E., Marschner H. (1991). Extension of the phosphorus depletion zone in VA-mycorrhizal white clover in a calcareous soil. Plant Soil.

[9] Li X.L., George E., Marschner H. (1991). Phosphorus depletion and pH decrease at the root soil and hyphae soil interfaces of VA mycorrhizal white clover fertilized with ammonium. New Phytol..

[10] Yao Q., Li X.L., Feng G., Christie P. (2001). Mobilization of sparingly soluble inorganic phosphates by the external mycelium of an abuscular mycorrhizal fungus. Plant Soil.

[11] Alewell C., Ringeval B., Ballabio C., Robinson D.A., Panagos P., Borrelli P. (2020). Global phosphorus shortage will be aggravated by soil erosion. Nat. Commun..

[12] Sattari S.Z., Bouwman A.F., Rodriguez R.M., Beusen A.H.W., van Ittersum M.K. (2016). Negative global phosphorus budgets challenge sustainable intensification of grasslands. Nat. Commun..

[13] Tonini D., Saveyn H.G.M., Huygens D.J.N.S. (2019). Environmental and health co-benefits for advanced phosphorus recovery. Nat. Sustain..

[14] Tisserant E., Malbreil M., Kuo A., Kohler A., Symeonidi A., Balestrini R., Charron P., Duensing N., Frei d.F.N., Gianinazzi-Pearson V. (2013). Genome of an arbuscular mycorrhizal fungus provides insight into the oldest plant symbiosis. Proc. Natl. Acad. Sci. USA.

[15] Morin E., Miyauchi S., San C.H., Chen E.C.H., Pelin A., de La Providencia I., Ndikumana S., Beaudet D., Hainaut M., Drula E. (2019). Comparative genomics of *Rhizophagus irregularis*, *R. cerebriforme*, *R. diaphanus* and *Gigaspora rosea* highlights specific genetic features in Glomeromycotina. New phytol..

[16] Miyauchi S., Kiss E., Kuo A., Drula E., Kohler A., Sánchez-García M., Morin E., Andreopoulos B., Barry K.W., Bonito G. (2020). Large-scale genome sequencing of mycorrhizal fungi provides insights into the early evolution of symbiotic traits. Nat. Commun..

[17] Jiang F., Zhang L., Zhou J., George T.S., Feng G. (2021). Arbuscular mycorrhizal fungi enhance mineralisation of organic phosphorus by carrying bacteria along their extraradical hyphae. New Phytol..

[18] Wang L., Zhang L., George T.S., Feng G. (2023). A core microbiome in the hyphosphere of arbuscular mycorrhizal fungi has functional significance in organic phosphorus mineralization. New Phytol..

[19] Duan S., Declerck S., Feng G., Zhang L. (2023). Hyphosphere interactions between *Rhizophagus irregularis* and *Rahnella aquatilis* promote carbon-phosphorus exchange at the peri-arbuscular space in *Medicago truncatula*. Environ. Microbiol..

[20] Emmett B.D., Lévesque-Tremblay V., Harrison M.J. (2021). Conserved and reproducible bacterial communities associate with extraradical hyphae of arbuscular mycorrhizal fungi. ISME J..

[21] Toljander J.F., Lindahl B.D., Paul L.R., Elfstrand M., Finlay R.D. (2007). Influence of arbuscular mycorrhizal mycelial exudates on soil bacterial growth and community structure. FEMS Microbiol. Ecol..

[22] Bharadwaj D.P., Alström S., Lundquist P.O. (2012). Interactions among *Glomus irregulare*, arbuscular mycorrhizal spore-associated bacteria, and plant pathogens under in vitro conditions. Mycorrhiza.

[23] Luthfiana N., Inamura N., Tantriani, Sato T., Saito K., Oikawa A., Chen W., Tawaraya K. (2021). Metabolite profiling of the hyphal exudates of *Rhizophagus clarus* and *Rhizophagus irregularis* under phosphorus deficiency. Mycorrhiza.

[24] Hildebrandt U., Janetta K., Bothe H. (2002). Towards growth of arbuscular mycorrhizal fungi independent of a plant host. Appl. Environ. Microbiol..

[25] Hildebrandt U., Ouziad F., Marner F.J., Bothe H. (2006). The bacterium *Paenibacillus validus* stimulates growth of the arbuscular mycorrhizal fungus *Glomus intraradices* up to the formation of fertile spores. FEMS Microbiol. Lett..

[26] Görke B., Stülke J. (2008). Carbon catabolite repression in bacteria: Many ways to make the most out of nutrients. Nat. Rev. Microbiol..

[27] Zhalnina K., Louie K.B., Hao Z., Mansoori N., da Rocha U.N., Shi S., Cho H., Karaoz U., Loqué D., Bowen B.P. (2018). Dynamic root exudate chemistry and microbial substrate preferences drive patterns in rhizosphere microbial community assembly. Nat. Microbiol..

[28] Baudoin E., Benizri E., Guckert A. (2003). Impact of artificial root exudates on the bacterial community structure in bulk soil and maize rhizosphere. Soil Biol. Biochem..

[29] Blagodatskaya E., Kuzyakov Y. (2008). Mechanisms of real and apparent priming effects and their dependence on soil microbial biomass and community structure: Critical review. Biol. Fertil. Soils.

[30] Eilers K.G., Lauber C.L., Knight R., Fierer N. (2010). Shifts in bacterial community structure associated with inputs of low molecular weight carbon compounds to soil. Soil Biol. Biochem..

[31] Zhang L., Peng Y., Zhou J., George T.S., Feng G. (2020). Addition of fructose to the maize hyphosphere increases phosphatase activity by changing bacterial community structure. Soil Biol. Biochem..

[32] Gralka M., Pollak S., Cordero O.X. (2023). Genome content predicts the carbon catabolic preferences of heterotrophic bacteria. Nat. Microbiol..

[33] Faghihinia M., Jansa J., Halverson L.J., Staddon P.L. (2023). Hyphosphere microbiome of arbuscular mycorrhizal fungi: A realm of unknowns. Biol. Fertil. Soils.

[34] Janda J.M., Abbott S.L. (2007). 16S rRNA gene sequencing for bacterial identification in the diagnostic laboratory: Pluses, perils, and pitfalls. J. Clin. Microbiol..

[35] Ragot S.A., Kertesz M.A., Bünemann E.K. (2015). *PhoD* alkaline phosphatase gene diversity in soil. Appl. Environ. Microbiol..

[36] Wang F., Shi N., Jiang R., Zhang F., Feng G. (2016). In situ stable isotope probing of phosphate-solubilizing bacteria in the hyphosphere. J. Exp. Bot..

[37] Trouvelot A., Gianinazzi-Pearson V., Gianinazzi S. (1986). Mesure du taux de mycorhization VA d’un systeme radiculaire. Recherche de methodes d’estimation ayant une significantion fonctionnelle. Mycorrhizae: Physiology and Genetics.

[38] Olsen S.R., Cole C., Watanabe C.V., Dean L.A. (1954). Estimation of Available Phosphorus in Soils by Extraction with Sodium Bicarbonate.

[39] Schoenau J.J., Huang W.Z. (1991). Anion-exchange membrane, water, and sodium-bicarbonate extractions as soil tests for phosphorus. Soil Sci. Plant Anal..

[40] Tabatabai M.A., Bremner J.M. (1969). Use of *p*-nitrophenyl phosphate for assay of soil phosphatase activity. Soil Biol. Biochem..

[41] Stubner S. (2002). Enumeration of 16S rDNA of desulfotomaculum lineage 1 in rice field soil by real-time PCR with SybrGreen detection. J. Microbiol..

[42] Sakurai M., Wasaki J., Tomizawa Y., Shinano T., Osaki M. (2008). Analysis of bacterial communities on alkaline phosphatase genes in soil supplied with organic matter. Soil Sci. Plant Nutr..

[43] Bokulich N.A., Kaehler B.D., Rideout J.R., Dillon M., Bolyen E., Knight R., Huttley G.A., Gregory C.J. (2018). Optimizing taxonomic classification of marker-gene amplicon sequences with QIIME 2′s q2-feature-classifier plugin. Microbiome.

[44] Martin M.J.E.J. (2011). Cutadapt removes adapter sequences from high-throughput sequencing reads. EMBnet. J..

[45] Callahan B.J., McMurdie P.J., Rosen M.J., Han A.W., Johnson A.J., Holmes S.P. (2016). DADA2: High-resolution sample inference from Illumina amplicon data. Nat. Methods.

[46] Katoh K., Misawa K., Kuma K., Miyata T. (2002). MAFFT: A novel method for rapid multiple sequence alignment based on fast Fourier transform. Nucleic Acids Res..

[47] Quast C., Pruesse E., Yilmaz P., Gerken J., Schweer T., Yarza P., Peplies J., Glöckner F.O. (2013). The SILVA ribosomal RNA gene database project: Improved data processing and web-based tools. Nucleic Acids Res..

[48] Zheng B., Zhu Y., Sardans J., Peñuelas J., Su J. (2018). QMEC: A tool for high-throughput quantitative assessment of microbial functional potential in C, N, P, and S biogeochemical cycling. Sci. Chin. Life Sci..

[49] Looft T., Johnson T.A., Allen H.K., Bayles D.O., Alt D.P., Stedtfeld R.D., Sul W.J., Stedtfeld T.M., Chai B., Cole J.R. (2012). In-feed antibiotic effects on the swine intestinal microbiome. Proc. Natl. Acad. Sci. USA.

[50] Zhu Y.G., Zhao Y., Li B., Huang C.L., Zhang S.Y., Yu S., Chen Y.S., Zhang T., Gillings M.R., Su J.Q. (2017). Continental-scale pollution of estuaries with antibiotic resistance genes. Nat. Microbiol..

[51] Graham J.H. (2001). What do root pathogens see in mycorrhizas?. New Phytol..

[52] Zhang L., Zhou J., George T.S., Limpens E., Feng G. (2022). Arbuscular mycorrhizal fungi conducting the hyphosphere bacterial orchestra. Trends Plant Sci..

[53] Wang F., Zhang L., Zhou J., Rengel Z., George T.S., Feng G. (2022). Exploring the secrets of hyphosphere of arbuscular mycorrhizal fungi: Processes and ecological functions. Plant Soil.

[54] Rozmoš M., Bukovská P., Hršelová H., Kotianová M., Dudáš M., Gančarčíková K., Jansa J. (2022). Organic nitrogen utilisation by an arbuscular mycorrhizal fungus is mediated by specific soil bacteria and a protist. ISME J..

[55] Dai Z., Liu G., Chen H., Chen C., Wang J., Ai S., Wei D., Li D., Ma B., Tang C. (2020). Long-term nutrient inputs shift soil microbial functional profiles of phosphorus cycling in diverse agroecosystems. ISME J..

[56] Ruvindy R., Barua A., Bolch C.J.S., Sarowar C., Savela H., Murray S.A. (2023). Genomic copy number variability at the genus, species and population levels impacts in situ ecological analyses of dinoflagellates and harmful algal blooms. ISME Commun..

