# Supplementary material for: Potential Role of Sugars in the Hyphosphere of Arbuscular Mycorrhizal Fungi to Enhance Organic Phosphorus Mobilization
Source: J Fungi (Basel). 2024 Mar 20;10(3):226. doi: 10.3390/jof10030226 (PMC10970774; doi:10.3390/jof10030226)
Supplement: Supplementary file 1 [file jof-10-00226-s001.zip › jof-2848780-supplementary.pdf]

Supplementary Materials

Potential Role of Sugars in the Hyphosphere of Arbuscular Mycorrhizal Fungi to Enhance Organic Phosphorus Mobilization

Zexing Jin [1,†], Guiwei Wang [1,2,†], Timothy S. George [3] and Lin Zhang [1,*]

[1] State Key Laboratory of Nutrient Use and Management, Key Laboratory of Plant-Soil Interactions, College of Resources and Environmental Sciences, Ministry of Education, China Agricultural University, Beijing 100193, China; 15129310266@163.com (Z.J.); gwwang2019@163.com (G.W.)
[2] National Engineering Research Center for Efficient Utilization of Soil and Fertilizer Resources, National Engineering and Technology Research Center for Slow and Controlled Release Fertilizers, College of Resources and Environment, Shandong Agricultural University, Taian 271018, China
[3] The James Hutton Institute, Invergowrie, Dundee DD2 5DA, UK; tim.george@hutton.ac.uk
* Correspondence: linzhang@cau.edu.cn; Tel.: +86-10-62733886; Fax: +86-10-62731016
† These authors contributed equally to this work.

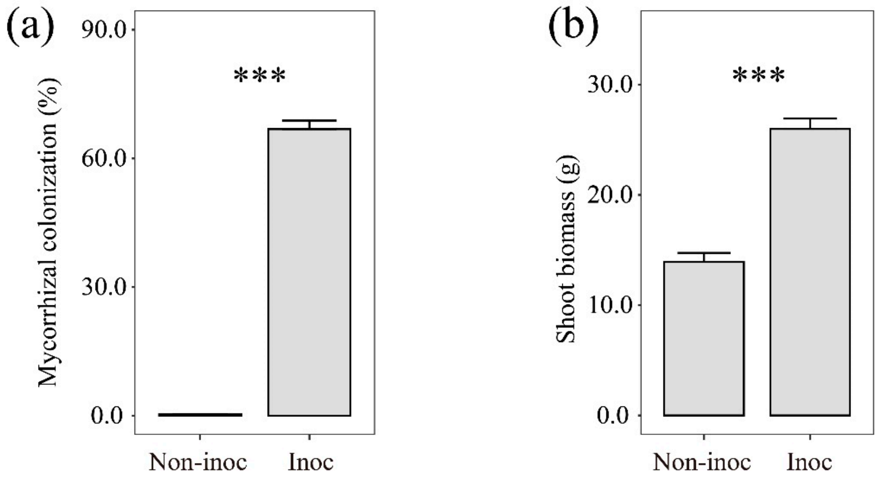

Figure S1 (a) Mycorrhizal colonization of the soil in hyphal compartments in AM fungus-inoculated pots. (b) Shoot biomass of maize plants in AM fungus-inoculated or non-inoculated pots. The asterisks indicate significant differences between the two treatments (*t*-test, $p < 0.001$). Non-inoc, non-inoculated pots. Inoc, AM fungus-inoculated pots.

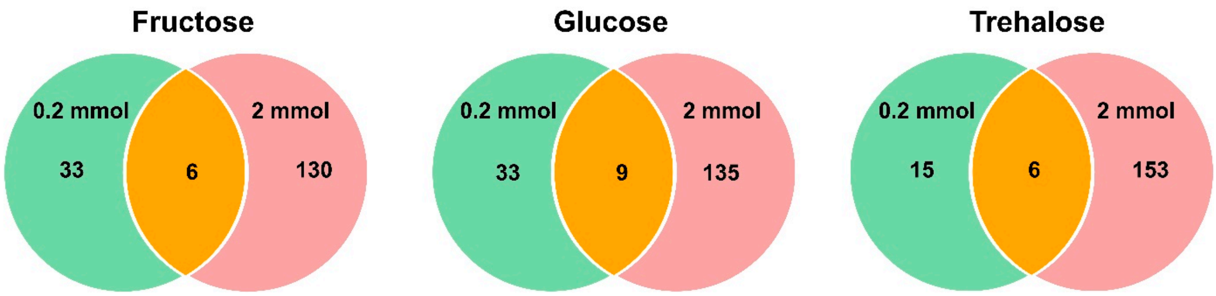

Figure S2 Venn plot showing the number of ASVs enriched in the hyphal compartments upon the three sugars' addition with two concentrations compared to non-sugar addition in non-inoculated pots. Green represents the number of ASVs enriched at 0.2 mmol C kg^{-1} soil compared to 0 mmol C kg^{-1} soil. Red represents the number of ASVs enriched at 2 mmol C kg^{-1} soil compared to 0 mmol C kg^{-1} soil. Orange represents the number of the same ASVs enriched at both 0.2 mmol C kg^{-1} soil and 2 mmol C kg^{-1} soil.

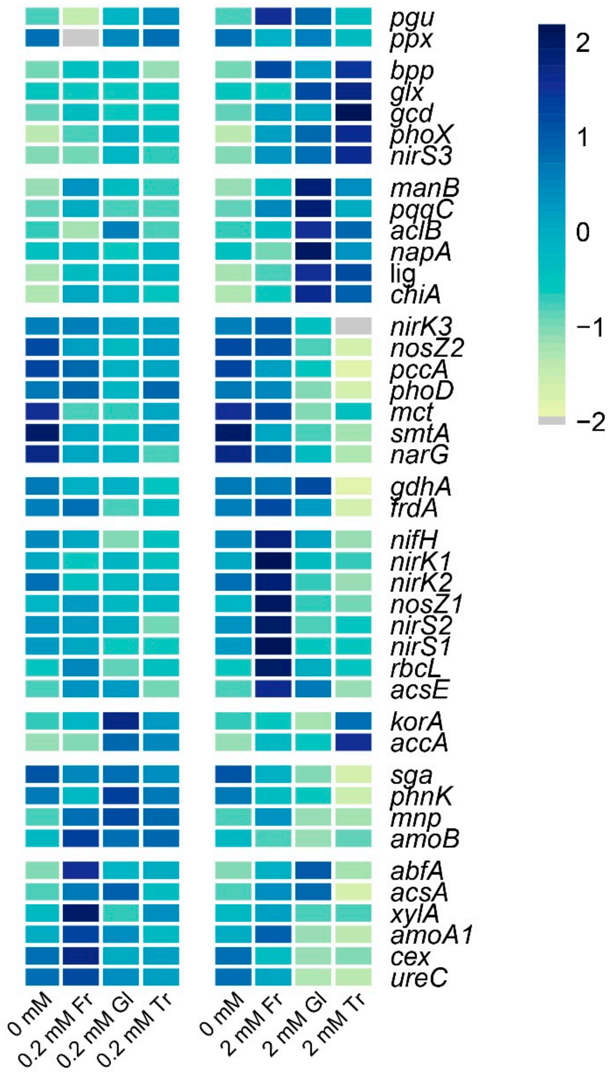

Figure S3 Heatmap of SmartChip profile, relative abundance of carbon (C), nitrogen (N) and phosphorus (P) turnover functional genes in the hyphal compartments upon three forms of sugar addition with three added concentrations (0, 0.2, 2 mmol C kg^{-1} soil) in non-inoculated pots. The values shown represent the mean relative abundance (n=4) of genes in the respective sugar addition. Fr: fructose, Gl: glucose, Tr: trehalose.

Table S1 The topological properties of co-occurrence networks in hyphal compartments after the addition of three forms of sugars

	**Fructose**	**Glucose**	**Trehalose**
**Total nodes**	1485	1231	1254
**Total edges**	3396	2415	2768
**Average Connectivity**	4.574	3.924	4.41
**Average clustering coefficient**	0.577	0.585	0.650
**Relative Modularity**	0.784	0.875	0.777
**Average path length**	11.019	13.164	9.826
**Graph diameter**	25.345	38.026	24.140

Table S2 Classification of functional genes

**Gene name**	**Gene function**
chiA, Lig, Glx, xylA, abfA, manB, mnp, cex, sga, pgu	carbon degradation
aclB, accA, rbcL, acsA, mct, smtA, korA, pccA, frdA, acsE	carbon sequestration
ppx, bpp, phoX, phnK, phoD, pqqC, gcd, gdhA	phosphorus cycle
amoA1, napA, nirS3, nirK1, nirK2, amoB, nirS2, ureC, narG, nosZ2, nirK3, nosZ1, nirS1, nifH	nitrogen cycle
